# Deciphering *Staphylococcus sciuri* SAT-17 Mediated Anti-oxidative Defense Mechanisms and Growth Modulations in Salt Stressed Maize (*Zea mays* L.)

**DOI:** 10.3389/fmicb.2016.00867

**Published:** 2016-06-09

**Authors:** Muhammad S. Akram, Muhammad Shahid, Mohsin Tariq, Muhammad Azeem, Muhammad T. Javed, Seemab Saleem, Saba Riaz

**Affiliations:** ^1^Department of Botany, Government College UniversityFaisalabad, Pakistan; ^2^Department of Bioinformatics and Biotechnology, Government College UniversityFaisalabad, Pakistan

**Keywords:** antioxidants, biofertilizer, reactive oxygen species, salinity, *Staphylococcus sciuri*, Zea mays

## Abstract

Soil salinity severely affects plant nutrient use efficiency and is a worldwide constraint for sustainable crop production. Plant growth-promoting rhizobacteria, with inherent salinity tolerance, are able to enhance plant growth and productivity by inducing modulations in various metabolic pathways. In the present study, we reported the isolation and characterization of a salt-tolerant rhizobacterium from Kallar grass [*Leptochloa fusca* (L.) Kunth]. Sequencing of the 16S rRNA gene revealed its lineage to *Staphylococcus sciuri* and it was named as SAT-17. The strain exhibited substantial potential of phosphate solubilization as well as indole-3-acetic acid production (up to 2 M NaCl) and 1-aminocyclopropane-1-carboxylic acid deaminase activity (up to 1.5 M NaCl). Inoculation of a rifampicin-resistant derivative of the SAT-17 with maize, in the absence of salt stress, induced a significant increase in plant biomass together with decreased reactive oxygen species and increased activity of cellular antioxidant enzymes. The derivative strain also significantly accumulated nutrients in roots and shoots, and enhanced chlorophyll and protein contents in comparison with non-inoculated plants. Similar positive effects were observed in the presence of salt stress, although the effect was more prominent at 75 mM in comparison to higher NaCl level (150 mM). The strain survived in the rhizosphere up to 30 days at an optimal population density (ca. 1 × 10^6^ CFU mL^-1^). It was concluded that *S. sciuri* strain SAT-17 alleviated maize plants from salt-induced cellular oxidative damage and enhanced growth. Further field experiments should be conducted, considering SAT-17 as a potential bio-fertilizer, to draw parallels between PGPR inoculation, elemental mobility patterns, crop growth and productivity in salt-stressed semi-arid and arid regions.

## Introduction

The world has experienced an exponential increase in population within the last few decades leading to a reduced availability of quality food. The problem is worsened due to an increase in the salinization of agricultural lands (Ghafoor et al., 2002). High levels of soluble salt in soil cause deleterious effects on germination, seedling vigor, crop establishment, plant metabolism and reproductive growth (Zhu et al., 2004), which contribute to a reduced yield of agronomically important crops. Exposure of plants to high salinity stress inhibits water uptake by roots and also induces osmotic shock, which modulates cell division, cell expansion and stomatal closure (Flowers, 2004). Long-term exposure to salts causes the increased uptake of Na^+^ together with a decrease in the uptake of Ca^2+^ and K^+^ (Yildirim et al., 2006). Nutritional imbalances/deficiencies result in the senescence of leaves, reducing photosynthetic area necessary to maintain the optimum growth. In addition, uptake and accumulation of Cl^-^ may disrupt photosynthetic function through the inhibition of nitrate reductase activity (Xu et al., 1999). Once the capacity of cells to store salts is exhausted, salts build up in the intercellular space, which results in cell dehydration and death (Sheldon et al., 2004). Moreover, at higher salinity, plants ultimately die due to reduced leaf production and expansion rates caused by oxidative damage (Kravchik and Bernstein, 2013). In Pakistan, 8.6 million hectares of arable land is saline and high yields of crops are usually not acquired because a great amount of time and energy is annually spent on reclamation strategies.

A possible strategy to cope with the low productivity of saline lands is microbial assisted amelioration of salt-induced damage (Dodd and Pérez-Alfocea, 2012). Among soil microbiota, plant growth-promoting rhizobacteria (PGPR) are potential candidates that are capable of colonizing the rhizosphere, penetrating the roots and triggering plant salinity-tolerance mechanisms (Tank and Saraf, 2010; Sheng et al., 2011). They influence plant physiology by releasing growth regulators, 1-aminocyclopropane-1-carboxylic acid (ACC) deaminase activity, enhanced soil phosphate solubilization and up-regulating the conserved salinity responsive mechanisms (Rajput et al., 2013; Kim et al., 2014). Many PGPR with inherent salt-tolerance have been isolated, characterized and applied to plants to increase crop productivity in saline regions (Rajput et al., 2013; Ahmad et al., 2014). There is evidence that PGPR regulate hormonal status (Sahoo et al., 2014) and initiate antioxidant defense mechanisms in plants exposed to high salt stress (Islam et al., 2015). Members of the *Staphylococcus* genus have been isolated from diverse environments and characterized as having salt-tolerance potential (Roohi et al., 2012; Nanjani and Soni, 2014). It has been reported that *Staphylococcus* mitigated the deleterious effects of salinity in radish (Yildirim et al., 2008), sweet cherry (Zhou et al., 2015) and strawberry (Karlidag et al., 2013). Sagar et al. (2012) reported that *Staphylococcus arlettae* strain Cr11 promoted plant growth via the reduction of hexavalent chromium.

Besides up-regulating stress responsive factors, PGPR also enhance the mobilization of fixed nutrients in salt-affected soils (Paul and Lade, 2014). A major soil-fixed nutrient is phosphorus (P) which is bound to cations (Ca^2+^, Al^2+^, Fe^2+^) and thus remains unavailable to plants (Bhattacharyya and Jha, 2012). Farmers apply phosphate-based fertilizers where a very limited amount is used by the plants and a large amount of fertilizers are converted into insoluble complexes in the soil (Zaidi et al., 2009). The excessive use of fertilizers adversely affects the environment as these are a potential source of environmental contamination (Savci, 2012). Moreover, phosphate fertilizers often leach from the soil and cause the eutrophication of surface and groundwater sources (Sharpley, 1999; He et al., 2003). Alternatively, a trend toward the use of slow-release phosphate (rock phosphate) fertilizers has been reported (Duponnois et al., 2005). Furthermore, efforts have also been made to explore PGPR as fertilizer supplements with the objective of substantially reducing the use of synthetic fertilizers (Hanif et al., 2015; Shahid et al., 2015). The issues of low nutrient availability and oxidative damage in saline lands severely affect the growth and physiology of Maize (*Zea mays* L.), which is one of the important domesticated cereal crops grown widely throughout the world. It is a nutritious rich source of human food and animal feed, and also provides raw material for industrial products.

The present work was designed to explore the potential of a salt-tolerant PGPR strain *S. sciuri* SAT-17 to boost maize growth under saline environments. To the best of our knowledge, this is the first report elucidating the physiological and phenotypic responses of maize after inoculation with a phyto-beneficial *S. sciuri* strain. The study will raise attention toward the establishment of long-term programs involving PGPR-based bio-formulations for the efficient utilization of salt-affected soils.

## Materials and Methods

### Sampling Site and Bacterial Isolation

The roots and rhizospheric soil surrounding Kallar grass [*Leptochloa fusca* (L.) Kunth] was collected from salt rich fields located at/near Pakka Anna (31°13′60 N and 72°48′0 E), Punjab, Pakistan. The samples were transported to the laboratory in sterilized polythene bags. The roots were shaken gently in sterile distilled water to remove the loosely adhering soil. One gram of strictly adhered soil was added in 9 mL of 0.85% (w/v) NaCl solution and serially diluted, as described by Somasegaran and Hoben (1994). An amount of 100 μL from three dilutions (10^-4^, 10^-5^, and 10^-6^) was spread on nutrient agar, amended with 8% NaCl, and plates were incubated at 28 ± 2°C for 48 h. Purification of the culture was achieved through repeated-streaking and pure culture (designated as SAT-17) was stored in 20% (v/v) glycerol at -80°C. Colony morphology, cell shape, motility and Gram’s reaction was performed under a light microscope (Olympus, Tokyo, Japan) as described earlier (Vincent, 1970). Catalase (CAT) activity was determined by pouring H_2_O_2_ on the culture on a glass slide. The physical and chemical analysis of rhizospheric and bulk soil samples was carried out at Ayub Agriculture Research Institute, Faisalabad, Punjab, Pakistan.

### Molecular Identification and Phylogenetic Analysis

Total genomic DNA of the isolate “SAT-17” was isolated by the alkaline lysis method (Maniatis et al., 1982), quantified by the NanoDrop^TM^ 2000/2000c (Thermo Fisher Scientific, Waltham, MA, USA) and used to amplify the 16S rRNA gene using primers fD1 (5′ AGAGTTTGATCCTGGCTCAG 3′) and rD1 (5′ AAGGAGGTGATCCAGCC 3′) (Weisburg et al., 1991). The reaction mixture and thermocycler conditions were set as described earlier by Shahid et al. (2015). Subsequently, the amplicon was cloned in pTZ57R/T and sequencing of the 16S rRNA gene was carried out by Macrogen, South Korea. Trimming of raw sequences, BLASTn analysis and phylogenetic studies were conducted using the methods and software packages described previously by Shahid et al. (2016).

### Characterization of Salt-Tolerance and Plant-Beneficial Traits

#### Salt-Tolerance Studies

The pure culture of SAT-17 was streaked on nutrient agar media with various NaCl concentrations (0, 0.5, 1, 1.5, 2, 2.5, 3, 3.5, or 4 M) and the salt-tolerance level was determined by measuring the minimum inhibitory concentration (MIC) of NaCl. Additionally, SAT-17 was re-exposed to NaCl concentrations (0, 0.5, 1, 1.5, 2, 2.5 M) in nutrient broth and subjected to the serial dilution method (Somasegaran and Hoben, 1994) to determine the bacterial cell density up to the MIC.

#### Phosphate Solubilization

A single purified colony of isolate SAT-17 was inoculated in 100 mL Pikovskaya’s broth (Pikovskaya, 1948) medium supplemented with different levels of NaCl (0, 0.5, 1, 1.5, 2 or 2.5 M) and incubated at 30 ± 2°C for 240 h in an orbital shaker (150 rpm). Twenty mililiter of bacterial culture was harvested and centrifuged at 13,000 *g* for 10 min. The quantitative measurement of phosphate solubilization was performed according to phosphomolybdate blue color method (Murphy and Riley, 1962) using a UV-visible spectrophotometer (Shimadzu UV/VIS, Kyoto, Japan) at 882 nm.

#### Indole-3-Acetic Acid (IAA) Production

The method described by Gordon and Weber (1951) was used to estimate IAA synthesis potential of the strain SAT-17. A single colony of SAT-17 was inoculated to 100 mL nutrient broth media with (100 mg L^-1^) or without tryptophan, each amended with NaCl (0, 0.5, 1, 1.5, 2, or 2.5 M). The cultures were grown on an orbital shaker (150 rpm) at 30 ± 2°C for 48 h. Thereafter, the cultures were harvested and centrifuged at 13,000 *g*. An amount of 1 mL supernatant was mixed in 2 mL of Salkowisk’s reagent. The tubes were kept in the dark for 30 min for color development. Quantification was carried out through a spectrophotometer at 540 nm. The IAA solutions (0, 5, 10, 50, 100, 200, or 500 μg mL^-1^) were used to draw standard curve for comparative measurements.

#### 1-Aminocyclopropane-1-Carboxylic Acid (ACC) Deaminase Activity

The ability of isolate SAT-17 to use ACC as a sole nitrogen source was assessed in 5 mL DF salt minimal medium (Penrose and Glick, 2003) containing 3 μL of 0.5 M ACC and supplemented with different concentrations (0, 0.5, 1, 1.5, 2, or 2.5 M) of NaCl. Cultures were grown at 30 ± 2°C for 24 h in a shaker. To determine ACC deaminase activity, the turbidity of the inoculated cultures was compared to that of the non-inoculated control.

### Greenhouse Experiment

#### Comparative Fitness Studies and Inoculum Preparation

Rifampicin-resistant derivatives of strain SAT-17 (SAT-17_rif_) were constructed, followed by comparative growth studies with its wild-type (SAT-17_w_) as described earlier by Shahid et al. (2012). For inoculum preparation, SAT-17_rif_ was grown up to 10^9^ CFU mL^-1^ cell density. The culture was centrifuged at 8,000 *g* and washed twice with ddH_2_O. The cells were re-suspended in equal volume of ddH_2_O and diluted to 10^8^ CFU mL^-1^.

#### Experimental Soil

The soil with textural class clay loam (available P: 8.3 mg kg^-1^, total N: 0.89 g kg^-1^, available K: 112 mg kg^-1^, organic matter 1.6% and pH 7.1) was obtained from the botanical garden of Government College University, Faisalabad, Pakistan. The soil was pre-inoculated by mixing 7 mL of SAT-17_rif_ inoculum per 100 g of soil (inoculated soil) or by mixing 7 mL of ddH_2_O per 100 g of soil (non-inoculated soil). A total of 18 pots were filled (each with 600 g soil) where nine pots received the inoculated and nine the non-inoculated soil.

#### Plant Material and Experimental Design

Maize seeds (FH-992) were surface-sterilized by immersing in 5% (w/v) sodium hypochlorite for 10 min and subsequently washed thrice with ddH_2_O. The seeds were submerged in SAT-17_rif_ inoculum and ddH_2_O separately for 20 min. The SAT-17_rif_-inoculated seeds were sown in pots containing inoculated soil, while ddH_2_O-dipped seeds were sown in non-inoculated pots. Seed rate was set at eight seeds per pot. The plants were thinned to five plants per pot after seedling emergence. Initially, the pots were irrigated, with a 3 days interval, using canal water. When seedlings emerged, these were watered periodically with equal volumes of half strength Hoagland solution (Arnon and Hoagland, 1940) with 3 NaCl levels, i.e., 0, 75 or 100 mM (Batool et al., 2013; Kim et al., 2014). The total treatments were named as follows:

Control_0_: non-inoculated soil with 0 mM NaCl
Control_75_: non-inoculated soil with 75 mM NaCl
Control_150_: non-inoculated soil with 150 mM NaCl
SAT-17_0_: SAT-17_rif_ inoculated soil with 0 mM NaCl
SAT-17_75_: SAT-17_rif_ inoculated soil with 75 mM NaCl
SAT-17_150_: SAT-17_rif_ inoculated soil with 150 mM NaCl

The experiment was conducted in a greenhouse (day/night temperature 25/20°C, light/dark periods 16/8) with a completely randomized design (CRD) and three replications for each treatment.

#### Bacterial Recovery and Growth Data

Before seed sowing, three random samples from inoculated and non-inoculated soil were serially diluted (Somasegaran and Hoben, 1994) and spread on nutrient-agar plates amended with (50 μg mL^-1^) rifampicin to determine initial soil population density of SAT-17_rif_. Thereafter, one plant from each inoculated and non-inoculated pot was randomly uprooted, at 10, 20, and 30 days after sowing (DAS) and a survival rate of SAT-17_rif_ was determined, as described by Shahid et al. (2012). Plant growth attributes (length as well as fresh and dry weights) were recorded for the remaining plants at 30 DAS.

### Analysis of Physiological Parameters and Nutrient Acquisition Patterns of Maize Plants

#### Lipid Peroxidation

The malondialdehyde (MDA) content of plant tissue was determined by thiobarbituric acid (TBA) reaction (Heath and Packer, 1968) to estimate the level of lipid peroxidation. The shoots homogenized with 0.1% trichloroacetic acid (TCA) were centrifuged and the supernatant was added to 20% TCA containing 0.5% TBA. The reaction mixture was heated (100°C) for 30 min and subsequently cooled to stop the reaction. The samples were centrifuged again and the absorbance of the supernatant was measured at 532 nm using Ultrospec 3000 (Biochrom Ltd, Cambridge, England) and adjusted for non-specific absorbance at 600 nm. The extinction coefficient was 155 mM cm^-1^.

#### Proline Content

The method of Bates et al. (1973) was used to determine the proline concentrations. Fresh leaves (ca. 0.5 g) were homogenized in sulfo-salicylic acid (3%, w/v) and the filtrate (2 mL) was mixed with 2 mL of acid ninhydrin reagent and 2 mL of glacial acetic acid. The mixture was incubated at 100°C for 60 min followed by cooling. Toluene (4 mL) was added to the solution and the contents were mixed well. The absorbance of the lower layer (chromophore-containing toluene) was observed spectrophotometrically at 520 nm.

#### Catalase Activity

Leaf material was homogenized in phosphate buffer (50 mM and pH 7.8) and centrifuged at 10,000 *g* for 10 min. The CAT activity of the supernatant was measured (Aebi, 1984) where the sample (ca. 100 μL) was mixed with H_2_O_2_ (0.75 M) and a decrease in absorbance was recorded at 240 nm for 20 s with the Ultrospec 3000 (Biochrom Ltd, Cambridge, England). Extinction coefficient was 0.039 mM cm^-1^.

#### Peroxidase (POD) Activity

The protocol described by Chance and Maehly (1955) with some modifications was used for the determination of POD activity. The plant samples were ground in phosphate buffer (pH 7.8) and centrifugation was performed at 8,000 *g* at 25°C. The absorbance of the reaction mixture [(2.7 mL phosphate buffer (pH 5), 0.1 mL Guiacol (20 mM), 0.1 mL H_2_O_2_ (40 mM) and 0.1 mL plant extract] was measured every 20 s for 2 min at 470 nm in a spectrophotometer. One unit of POD activity was considered as change of absorbance of 0.01 units min^-1^.

#### Total Phenolics Content

A 0.5 g of fresh leaf tissue was homogenized in 80% (v/v) acetone solution and centrifuged at 10,000 *g* for 10 min at 4°C. The supernatant (100 μL) was diluted with 2 mL of water plus 1 mL of Folin–Ciocalteau’s phenol reagent. Five mL of 20% (w/v) Na_2_CO_3_ was then added and the volume was made up to 10 mL with ddH_2_O. The absorbance was read at 750 nm and the results were expressed as mg g^-1^ FW of leaf (Julkunen-Tiitto, 1985) by comparison with standards of known concentrations.

#### Determination of Plant Nutrient Elements

The plant roots and shoots were oven-dried at 105°C for 24 h. The oven-dried ground plant material (ca. 0.5 g) was taken in digestion flasks containing 5 mL conc. H_2_SO_4_ (Wolf, 1982). The flasks were incubated overnight at room temperature. A 0.5 mL solution of 35% (v/v) H_2_O_2_ was poured and the flasks were then placed over a hot plate (350°C). The digestion flasks remained on a hot plate until no fumes were produced. Afterward, these were removed from the hot plate and allowed to cool. The step was repeated until the digestion mixture became fully transparent. The cooled mixtures were then diluted up to 50 mL, filtered and stored at 4°C. The N, P, K, Ca, and Mg contents of the dried shoot and root samples were determined using an atomic absorption spectrophotometer (Hitachi, Model 7JO-8024, Tokyo, Japan) with flame spectrophotometry. To minimize the matrix affect during plant metal analysis, standard reference materials (SRM) and standard solutions were used.

#### Plant Chlorophyll Content

The method of Arnon (1949) was used for the determination of plant chlorophyll contents. An 80% (v/v) solution of acetone was used for homogenization followed by centrifugation and filtration. The absorbance of the supernatant was recorded using a spectrophotometer at three different wavelengths, i.e., 663, 645, and 480 nm.

#### Plant Protein Content

Fresh plant material was homogenized, centrifuged (at 10,000 *g* for 15 min at 4°C) and mixed with Bradford reagent (Bradford, 1976). The mixture was incubated for 15–20 min and the absorbance was measured spectrophotometrically at 595 nm. Total soluble proteins were estimated by comparison with a standard curve of bovine serum albumin (BSA).

### Data Analysis

Data for green house experiments were analyzed statistically by analysis of variance (Steel et al., 1997) using Statistix (ver. 8.1) software. The least significant difference test (Fisher’s LSD) at 5% probability was used to compare the differences between treatment means. The phylogenetic tree was constructed with MEGA (ver. 6) software (Tamura et al., 2013).

## Results

### Soil Physicochemical Analysis

The physical and chemical properties of bulk and Kallar grass rhizospheric soil samples collected from Pakka Anna were determined and are presented in **Table 1**. Soil texture was sandy loam for bulk soil and sandy clay loam for rhizospheric soil. Both soils exhibited high EC and pH but were deficient in organic matter contents; this is an indication of saline nature. The examined soils were also low in total N and available P contents.

**Table 1:** Physicochemical properties of soil samples from Pakka Anna.

Soil properties	Bulk soil	Rhizospheric soil
Textural class	Sandy loam	Sandy clay loam
Sand (%)	62	61
Silt (%)	23	19
Clay (%)	15	20
Bulk density (mg m^-3^)	1.44	151
EC (dS m^-1^)	8.13	8.11
pH	8.5	7.6
Organic matter (%)	1.80	1.49
Organic C (g kg^-1^)	3.9	4.32
Total N (g kg^-1^)	0.49	0.56
C:N ratio	8	7.7
Available P (mg kg^-1^)	3.4	4.2
Available K (mg kg^-1^)	176	210

### Molecular Identification of SAT-17

**FIGURE 1:**
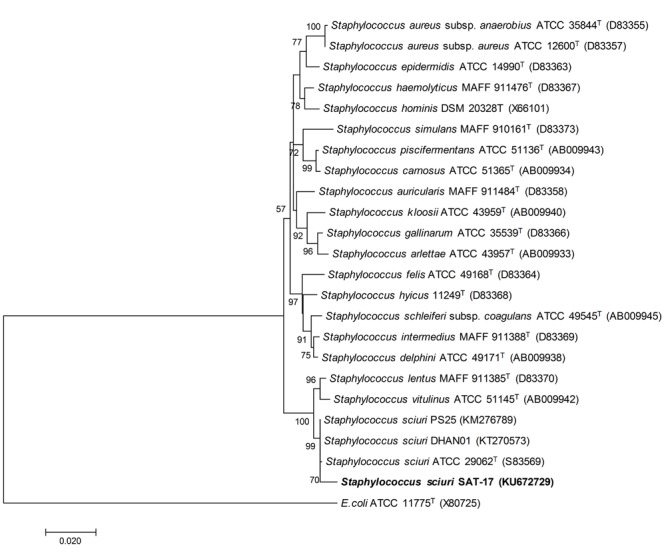
**Phylogenetic analysis of *S. sciuri* SAT-17 with type strains of genus *Staphylococcus* and the closest GenBank matches.** The evolutionary history was inferred using the Neighbor-Joining method. The optimal tree with the sum of branch length = 0.39351674 is shown. The percentages (≥50%) of replicate trees in which the associated taxa clustered together in the bootstrap test (1000 replicates) are shown. The evolutionary distances were computed using the Maximum Composite Likelihood method and are in the units of the number of base substitutions per site. Codon positions of 24 nucleotide sequences included were 1st + 2nd + 3rd + Non-coding.

The isolate was identified as *S. sciuri* SAT-17 on the basis of 16S rRNA gene sequence analysis. BLASTn analysis of 1388 bp sequence (submitted to NCBI GenBank as Acc. # KU672729) of SAT-17 showed 99% sequence identity with *S. sciuri* PS25 (Acc. # KM276789) and *S. sciuri* DHAN01 (Acc. # KT270573) (Supplementary Figure S1). Furthermore, strain SAT-17 was placed in a cluster of *S. sciuri* ATCC 29062^T^ (Acc. # S83569), *S. sciuri* PS25 (Acc. # KM276789) and *S. sciuri* DHAN01 (Acc. # KT270573) in a phylogenetic tree (**Figure 1**).

### Physiological Characterization

*Staphylococcus sciuri* SAT-17 was able to grow in culture medium amended with 2.5 M NaCl and this level of *in vitro* salinity stress was considered the MIC for the strain. Moreover, the bacterial cell density (Log CFU mL^-1^ 24 h^-1^) was measured in the following descending order at 0, 0.5, 1, 1.5, and 2 M NaCl concentration: 10.6 ± 0.7 > 10.5 ± 0.8 > 9.3 ± 0.4 > 7.2 ± 0.5 > 5.4 ± 0.3 > 2.9 ± 0.3 (**Table 2**).

**Table 2:** Physiological characterization of *S. sciuri* SAT-17 under salinity stress.

Parameters	NaCl concentration					
	0 M	0.5 M	1.0 M	1.5 M	2.0 M	2.5 M
Bacterial cell density (Log CFU mL^-1^ 24 h^-1^)	10.6 (0.7)	10.5 (0.8)	9.3 (0.4)	7.2 (0.5)	5.4 (0.3)	2.9 (0.3)
P solubilization (μg mL^-1^)	36.5 (4.7)	40.8 (5.2)	33.2 (3.7)	18.7 (2.5)	7.6 (1.1)	ND
IAA production (With tryptophan)	9.1 (0.49)	11.8 (0.76)	9.9 (0.91)	8.6 (0.51)	3.2 (0.23)	ND
IAA production (Without tryptophan)	16 (0.83)	17.6 (0.64)	11.9 (0.73)	ND	ND	ND
ACC deaminase activity	+	+	+	+	-	-

*Each value is the mean of three replicates and standard errors are presented in parentheses. ND, not determined.*

The tricalcium phosphate (TCP) solubilizing ability of the strain was found to be up to 40.8 ± 5.2 μg mL^-1^ in the presence of 0.5 M salt. This ability decreased together with an increase in salt level and only 7.6 ± 1.1 μg mL^-1^ TCP was solubilized at a concentration of 2 M NaCl. The strain synthesized IAA (3.2 ± 0.23 μg mL^-1^) up to 2 M NaCl addition in the presence of tryptophan, while in the absence of tryptophan, 11.9 ± 0.73 μg mL^-1^ IAA was measured up to 1.0 M NaCl stress. *S. sciuri* SAT-17 was also found to be positive for *in vitro* ACC deaminase activity with the addition of up to 1.5 M NaCl (**Table 2**).

### Comparative Growth Studies and Bacterial Recovery from Rhizosphere

**FIGURE 2:**
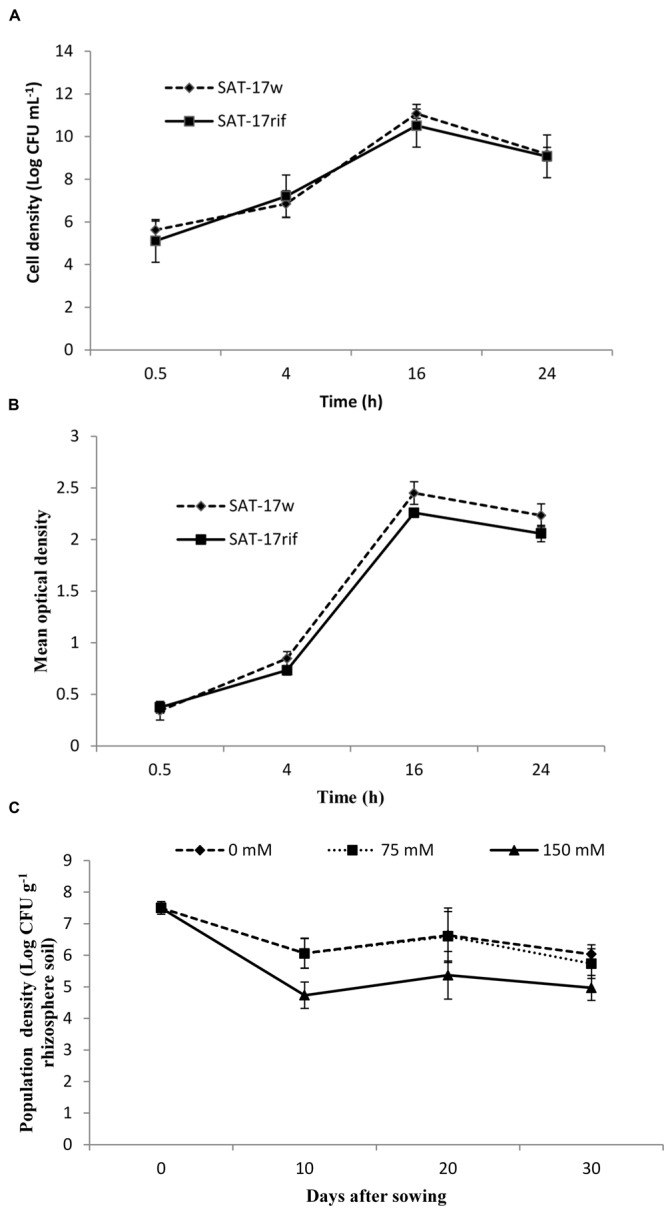
**Comparative growth curve of wild-type and rifampicin-resistant derivative of *S. sciuri* SAT-17 constructed with the plate-count method **(A)** and by measuring OD **(B)**.** Population density of *S. sciuri* SAT-17 recovered from maize rhizosphere at different time intervals **(C)**. Error bars represent standard errors (*n* = 3).

The wild type strain (SAT-17_w_) and its derivative (SAT-17_rif_) showed similar growth behaviors in plate count method and spectrophotometric OD methods, as presented in **Figure 2**. The growth of both SAT-17_w_ and SAT-17_rif_ was found to be optimal (ca. 1 × 10^10^ CFU mL^-1^) and a normal bacterial growth curve pattern was obtained after plotting the measurements obtained at different time intervals.

The population density of SAT-17_rif_, on rifampicin-amended agar plants before seed sowing, was found to be 3.5 × 10^7^ CFU g^-1^ of soil. The strain survived in the maize rhizosphere up to 30 DAS at an optimal population density of 6 × 10^6^ CFU g^-1^ soil (in treatment SAT-17_0_) and 4.9 × 10^5^ CFU g^-1^ soil (in treatment SAT-17_75_). The population density of SAT17_rif_ decreased to 8.9 × 10^4^ CFU g^-1^ in the treatment SAT-17_150_. We did not observe any bacterial growth on agar plates for the treatments control_0_, control_75_ and control_150_ (**Figure 2C**).

### Effect of Salt Stress and SAT-17 Inoculation on Maize Growth

In the absence of SAT-17 inoculation, a decrease in root length was observed with increasing salt level. The SAT-17 inoculation resulted in enhanced root length, with a maximum (9.98 cm) in the absence of NaCl. At 75 mM salt stress, the percentage difference between the SAT-17 treated and non-treated plants was 55.9%, where the treated ones exhibited greater root length (8.83 cm). Similarly, at 150 mM salt stress, we observed a percentage difference of 42.6% with higher root length in plants that received the treatment, i.e., SAT-17_150_ (**Table 3**).

**Table 3:** Effect of *S. sciuri* SAT-17 inoculation on various growth parameters of maize with or without salt stress.

Treatments^∗^	Root length (cm)	Shoot length (cm)	Root FW (g)	Shoot FW (g)	Root DW (g)	Shoot DW (g)
Control_0_	7.87 (0.66)^c∗∗^	26 (1.02)^cd^	4.73 (0.37)^d^	14.9 (1.05)^b^	1.23 (0.32)^b^	4.03 (0.68)^b^
Control_75_	4.97 (0.25)^e^	24.3 (1.52)^de^	3.71 (0.44)^e^	11.5 (0.80)^c^	0.97 (0.23)^b^	3.74 (0.54)^b^
Control_150_	4.37 (0.41)^e^	21.7 (1.15)^e^	3.10 (0.60)^f^	9.8 (1.30)^c^	0.81 (0.18)^b^	3.49 (0.50)^b^
SAT-17_0_	9.98 (0.55)^a^	31 ± (2)^a^	8.55 (0.18)^a^	18.7 (1.52)^a^	2.31 (0.61)^a^	6.34 (0.87)^a^
SAT-17_75_	8.83 (0.50)^b^	30.3 (2.30)^ab^	7.75 (0.31)^b^	19.3 (1.49)^a^	2.26 (0.80)^a^	6.31 (0.60)^a^
SAT-17_150_	6.74 (0.36)^d^	29.7 (3.21)^bc^	6.18 (0.41)^c^	15.3 (0.57)^b^	1.34 (0.56)^ab^	4.50 (0.45)^b^

*^∗^Control_0_, non-inoculated control without NaCl; Control_*75*_, non-inoculated control with 75 mM NaCl; Control_*150*_, non-inoculated control with 150 mM NaCl; SAT-17_0_, inoculation with *S. sciuri* SAT-17 without NaCl; SAT-17_*75*_, inoculation with *S. sciuri* SAT-17 with 75 mM NaCl; SAT-17_*150*_, inoculation with *S. sciuri* SAT-17 with 150 mM NaCl. ^∗^Data, analyzed by one-way analysis of variance, are presented as the mean of three replications (*n* = 3) and standard errors are presented in parentheses. Values that differ significantly (Fisher’s LSD; *P* ≤ 0.05) are presented with different lower-case letters and standard errors are given in parentheses.*

The control plants exhibited a shoot length of 26 cm and the addition of salt reduced the shoot length, significantly at 150 mM (19.2%). The inoculation with SAT-17 increased the shoot length both in the absence (0 mM) and presence (75 or 150 mM) of NaCl. The SAT-17 treatment, in the presence of 75 mM salt, resulted in a 24.6% increase in shoot length compared to the control, while, at this level of stress, there was 31.1% difference in shoot length between SAT-17 treated (29.7 cm shoot length) and non-treated (21.7 cm shoot length) plants (Supplementary Figure S3). The control plants and those grown in the presence of 150 mM salt, without SAT-17 treatment, exhibited 34% difference for the root fresh weight, where a lower weight was recorded in the latter group (3.1 g). The SAT-17 application increased the root FW, irrespective of the level of imposed stress. A similar trend was observed for root DW as well as shoot biomass.

### Effect of Salt Stress and SAT-17 Inoculation on Antioxidants

The control plants exhibited a phenolics content of 26.8 mg g^-1^ FW. When no SAT-17 treatment was applied, a reduction of 25 and 31.3% was observed, in comparison to the control, at 75 and 150 mM stress, respectively. When no salt stress was present, the SAT-17 treatment enhanced the content up to 50.3% (40.3 mg g^-1^ FW). Plants grown at a salt level of 75 mM along with SAT-17 treatment exhibited higher phenolic content (37.6 mg g^-1^ FW) compared to plants that received 75 mM salt stress but no SAT-17 inoculation (20.1 mg g^-1^ FW). There was a 42.8% difference between the SAT-17 treated and non-treated plants at 150 mM salt stress, with the former resulting in higher phenolics content (30.1 mg g^-1^ FW). Without SAT-17 application, we observed a 46.2% (at 75 mM salt) and 153% (at 150 mM salt) increase in the MDA content compared to the control plants (1.75 nmole/g^-1^ DW). The plants that received SAT-17 treatment, in the absence of salt, exhibited a 13.7% decrease in MDA content. At 75 mM salt stress, the MDA contents were 61.2% different in SAT-17 treated (1.3 nmole/g^-1^ DW) and non-treated plants (2.56 nmole/g^-1^ DW). A similar trend was observed at a salt level of 150 mM, where treated plants exhibited less MDA than the non-treated ones.

An H_2_O_2_ level of 13.8 ng g^-1^ DW was observed in the control plants. The presence of salt, without SAT-17_rif_ treatment, increased the H_2_O_2_ level, significantly (26.0%) at 150 mM salt stress. The plants that received 75 mM salt stress exhibited higher H_2_O_2_ levels (14.2 ng g^-1^ DW) than those which were grown with salt and SAT-17 treatment (12.5 ng g^-1^ DW). A similar trend was observed for plants grown with 150 mM salt stress alone and those that received salt stress as well as SAT-17 treatment. Without SAT-17 treatment, the maximum proline content (2.97 μg g^-1^ DW) was observed in plants grown with 150 mM salt level, with results that were 10.8 and 8.3% higher than for those plants grown with 0 and 75 mM salt levels, respectively. The application of SAT-17 (at 0 mM salt level) reduced the proline content in comparison to control plants, while at 75 mM, the SAT-17 treatment significantly increased (11.0%) the proline content in comparison to the plants that did not receive the treatment. There were no significant differences in proline content of the plants that received (2.96 μg g^-1^ DW) or did not receive (2.97 μg g^-1^ DW) SAT-17 treatment at the salt level of 150 mM. However, the observed values were significantly higher than those reported in the control plants (2.68 μg g^-1^ DW proline).

Without SAT17_rif_ inoculation, the 75 mM NaCl level increased the CAT activity (32.5 U/mg protein), while the higher stress level (150 mM) resulted in reduced (19.8 U/mg protein) CAT activity in comparison to control plants (28 U/mg protein). The imposition of salt stress (75 or 150 mM) combined with SAT-17 treatment (SAT-17_75_ and SAT-17_150_) resulted in higher CAT activity compared to the plants that received salt stress but not the SAT-17 application (Control_75_ and Control_150_). The maximum POD activity (38.2 U/mg protein) was observed in plants grown under 150 mM salt stress without SAT17_rif_ inoculation (control_150_ treatment). This POD activity was 70.5 and 101% higher than the activity observed in control_0_ (22.4 U/mg protein) and SAT-17_0_ (19.0 U/mg protein) plants, respectively. At 75 mM salt level, we observed a 9.9% difference in POD activity between the SAT-17 treated and non-treated plants. However, at the 150 mM salt level, the SAT-17 treated and non-treated plants exhibited a similar POD activity (**Table 4**).

**Table 4:** Oxidative stress responsive species and antioxidants as affected by inoculation of *S. sciuri* SAT-17 at different salinity stress levels.

Treatments^∗^	MDA (ηmol g^-1^ DW)	H_2_O_2_ (ηg g^-1^ DW)	Proline (μg g^-1^ DW)	CAT (U mg^-1^ protein)	POD (U mg^-1^ protein)	Total phenolics (mg g^-1^ FW)
Control_0_	1.75 (0.21)^c∗∗^	13.8 (1.50)^b^	2.68 (0.45)^a^	28 (2.54)^c^	22.4 (2.59)^c^	26.8 (1.05)^b^
Control_75_	2.56 (0.32)^b^	14.2 (0.36)^b^	2.74 (0.39)^a^	32.5 (1.44)^b^	37.1 (3.15)^a^	20.1 (2.24)^c^
Control_150_	4.43 (0.42)^a^	17.4 (0.75)^a^	2.97 (0.50)^a^	19.8 (2.83)^d^	38.2 (2.34)^a^	18.4 (2.98)^c^
SAT-17_0_	1.51 (0.12)^c^	10.4 (1.20)^d^	2.59 (0.12)^a^	26.7 (3.95)^c^	19.0 (3.54)^a^	40.3 (2.33)^a^
SAT-17_75_	1.36 (0.31)^c^	12.5 (1.38)^c^	3.08 (0.46)^a^	38.8 (3.80)^a^	33.6 (3.41)^b^	37.6 (1.23)^a^
SAT-17_150_	1.77 (0.17)^c^	11.9 (0.99)^c^	2.96 (0.40)^a^	38.9 (4.49)^a^	37.6 (2.18)^a^	30.1 (3.0)^b^

*^∗^Control_0_, non-inoculated control without NaCl; Control_*75*_, non-inoculated control with 75 mM NaCl; Control_*1*50_, non-inoculated control with 150 mM NaCl; SAT-17_*0*_, inoculation with *S. sciuri* SAT-17 without NaCl; SAT-17_*75*_, inoculation with *S. sciuri* SAT-17 with 75 mM NaCl; SAT-17_*150*_, inoculation with *S. sciuri* SAT-17 with 150 mM NaCl. ^∗∗^Data, analyzed by one-way analysis of variance, are presented as the mean of three replications (*n* = 3). Values that differ significantly (Fisher’s LSD; *P* ≤ 0.05) are presented with different lower-case letters and standard errors are given in parentheses.*

### Effect of Salt Stress and SAT-17 Inoculation on Maize Nutrient Physiology

The addition of NaCl hindered the uptake of soil N and a decrease in root N content was observed together with an increase in the imposed salt stress. Without SAT-17 treatment, a decrease of 13.6% in N content was observed in plants grown in the presence of 150 mM salt in comparison to control plants (30 mg g^-1^ DW). The SAT-17 treatment, at 75 mM salt, resulted in increased N content (30.5 mg g^-1^ DW) in comparison to plants that received only salt stress (Control_75_). However, at higher salt stress (150 mM), the difference between SAT-17 treated and non-treated plants was non-significant, with the former resulting in 26.1 mg g^-1^ DW and the latter 25.9 mg g^-1^ DW root N content. The SAT-17 treatment, in the absence or presence of salt stress, increased the shoot N content where the maximum shoot N content (28.7 mg g^-1^ DW) was observed in plants grown without salt stress (SAT-17_0_). At 75 mM stress, the difference between the SAT-17 treated and non-treated group was 9.64%, where the treated group showed higher shoot N. However, at a salt level of 150 mM, the difference between SAT-17 treated (SAT-17_150_) and non-treated (Control_150_) plants was not significant, with 20.2 mg g^-1^ DW and 19.6 mg g^-1^ DW shoot N, respectively (**Table 5**).

**Table 5:** Effect of *S. sciuri* SAT-17 inoculation on maize nutrient, chlorophyll and protein contents at different levels of salt-stress.

Treatments ^∗^	Root N (mg g^-1^ DW)	Shoot N (mg g^-1^ DW)	Root P (mg g^-1^ DW)	Shoot P (mg g^-1^ DW)	Root K (mg g^-1^ DW)	Shoot K (mg g^-1^ DW)	Total chlorophyll (mg g^-1^ FW)	Total protein (mg g^-1^ FW)
Control_o_	30 (1.20)^b∗∗^	26.7 (1.56)^b^	2.83 (0.3 l)^b^	2.2 (0.30)^b^	12.7 (l)^a^	9.8 (1.74)^a^	24.3 (3.19)^b^	374 (28)^c^
Control_75_	27.1 (2.28)^c^	22.7 (2.93)^d^	2.53 (0.55)^b^	2.07 (0.49)^b^	11.0 (0.90)^b^	8.7 (0.80)^b^	23 (3.97)^b^	254 (22)^d^
Control_150_	25.9 (1.42)^d^	19.6 (1.67)^e^	1.9 (0.15)^c^	0.98 (0.21)^d^	8.23 (0.70)^d^	6.00 (0.62)^c^	14.8 (2.88)^c^	217 (20)^d^
SAT-17_0_	32.8 (2.47)^a^	28.7 (1.93)^a^	3.50 (0.61)^a^	3.07 (0.55)^a^	13.1 (1.01)^a^	9.9 (0.56)^a^	39.3 (3.99)^a^	501 (32)^a^
SAT-17_75_	30.5 (3.26)^b^	25 (2.43)^c^	3.43 (0.77)^a^	3.06 (0.61)^a^	12.5 (0.66)^a^	10 (l)^a^	35.4 (2.39)^a^	432 (42)^b^
SAT-17_150_	26.1 (2.70)^d^	20.2 (1.68)^e^	2.16 (0.40)^b^	1.79 (0.40)^c^	9.2 (1.22)^c^	8.4 (1.90)^b^	28.4 (4.30)^b^	423 (26)^bc^

*^∗^Control_*0*_, non-inoculated control without NaCl; Control_*75*_, non-inoculated control with 75 mM NaCl; Control_*150*_, non-inoculated control with 150 mM NaCl; SAT-17_*0*_, inoculation with *S. sciuri* SAT-17 without NaCl; SAT-17_*75*_, inoculation with *S. sciuri* SAT-17 with 75 mM NaCl; SAT-17_*150*_, inoculation with *S. sciuri* SAT-17 with 150 mM NaCl. ^∗∗^Data, analyzed by one-way analysis of variance, are presented as the mean of three replications (*n* = 3). Values that differ significantly (Fisher’s LSD; *P* ≤ 0.05) are presented with different lower-case letters and standard errors are given in parentheses.*

The control plants exhibited 2.83 mg g^-1^ DW root P content. The addition of salt reduced the P uptake, significantly at the salt level of 150 mM (1.9 mg g^-1^ DW root P content). The SAT-17 treatment increased the uptake of P, at 0 and 75 mM NaCl levels, as indicated by higher root P content. At the higher salt level (150 mM), the reduction in P uptake was 2.16 mg g^-1^ DW and 1.9 mg g^-1^ DW with and without SAT-17 treatment, respectively. Without SAT-17 inoculation, the imposed salt stress reduced the shoot P content either non-significantly (at 75 mM salt) or significantly (at 150 mM salt) compared to control plants (2.2 mg g^-1^ DW shoot P content). The SAT-17 treatment increased the shoot P content, significantly, at all of the applied salt levels.

The root K contents were decreased together with an increase in the salt stress level, where 150 mM NaCl resulted in a 35.1% decrease in root K in comparison to the control (12.7 mg g^-1^ DW). The control plants exhibited 9.8 mg g^-1^ DW shoot K content. Without SAT-17 treatment, 150 mM salt stress resulted in a 38.7% decrease, while, at this level, the SAT-17 treated plants exhibited only a 14.2% decrease in comparison to control. The effect of strain SAT-17_rif_ inoculation on the uptake and translocation of Ca and Mg was found to be non-significant compared to non-inoculated plants exposed to different salinity levels (data not shown).

The control maize plants showed 24.3 mg g^-1^ FW total chlorophyll (Chl) content while SAT-17_rif_ inoculation, in the absence of salt, resulted in a 38.1% increase (39.3 mg g^-1^ FW). The salt stress, without SAT-17 application, resulted in a reduction of maize Chl contents, which was significant (64.1%) at 150 mM. A difference of 42.4% was observed between the treated and non-treated plants grown with 75 mM salt stress, where the former showed a higher Chl content (35.4 mg g^-1^ FW). A similar trend was observed between the treated and non-treated plants at a salt level of 150 mM.

## Discussion

The present work reported the identification and characterization of a PGPR strain isolated from the rhizosphere of a halophytic plant “Kallar grass [*Leptochloa fusca* (L.) Kunth].” The 16 s gene sequence identity (99%) and its clustering with *S. sciuri* ATCC 29062^T^ (S83569) (**Figure 1**) confirmed its molecular identity as *S. sciuri* and the strain was designated as SAT-17 (Supplementary Figure S2). Most strains of *S. sciuri* have been reported to form commensal associations with animals (Nemeghaire et al., 2014). Members of the genus *Staphylococcus* can tolerate high salt concentrations (Roohi et al., 2012; Khan et al., 2015) and exhibit plant growth-promoting properties (Yildirim et al., 2008). Zhou et al. (2015) reported the increased growth of sweet cherry plants after inoculation with a PGPR strain *S. sciuri* subspecies *sciuri*, grown in sterilized soil.

The *in vitro* phosphate solubilization ability of the strain was comparable with that reported previously (Oliveira et al., 2009). Solubilization of bound soil phosphate by PGPR triggered soil acidification by the production of organic acids, depending upon the number of carboxylic groups carried. The rhizospheric acidosis promoted the release of cations (Al^2+^, Fe^2+^, Ca^2+^) associated with phosphate, thereby making it available for plant uptake (Mullen, 2005; Trivedi and Sa, 2008). The strain SAT-17 produced a significant amount of IAA up to a salt treatment level of 2 M with the addition of tryptophan (**Table 2**). Spaepen et al. (2007) reported that IAA is one of the best characterized traits of many PGPR and it is an important phyto-hormone involved in the regulation of plant growth and development. The hormone is also thought to be involved in plant stress responses as a signaling molecule (Spaepen and Vanderleyden, 2011). The SAT-17 also exhibited ACC deaminase activity, a key factor for a PGPR strain to induce stress tolerance in host plants by controlling ethylene concentrations (Glick et al., 2007). Furthermore, the salt treatment level of 2.5 M was found to be the MIC for the isolate SAT-17, making it physiologically more competent than the previously reported PGPR strains.

The rifampicin-resistant derivatives of strain SAT-17 (*S. sciuri* SAT-17_rif_) were constructed so that the strain could successfully be recovered and identified after inoculation. Rifampicin was selected as a selectable marker due to the susceptibility of most soil bacteria against rifampicin (Shahid et al., 2012). The comparative growth curves of SAT-17_w_ and SAT-17_rif_ revealed that the derivative strain was healthy enough to inoculate the maize seedlings. It survived in the rhizosphere, with an optimal population density of 6 × 10^6^ CFU g^-1^ rhizospheric soil, up to 30 DAS, suggesting that the strain was rhizospherically competent (Shahid et al., 2012). Many PGPR exhibiting *in vitro* beneficial traits have failed to induce positive effects, *in vivo*, due to poor root colonization and antagonism with the native soil microorganisms (Benizri et al., 2001). The SAT-17_rif_ density was initially declined in the soil but later the strain adapted well and survived. Fischer et al. (2010) also reported an initial decrease in the density of the rifampicin-resistant derivative of *Pseudomonas* sp. SF4c in the rhizosphere of wheat which later became stable and induced beneficial effects.

Plant growth-promoting rhizobacteria can relieve plants from the deleterious effects of salinity and enhance plant growth and productivity through a variety of mechanisms (Rajput et al., 2013; Kim et al., 2014; Islam et al., 2015). *S. sciuri* SAT-17_rif_, when used as an inoculum, significantly promoted the growth and biomass of maize plants grown in the absence and presence of 75 mM salt stress, each compared with the non-inoculated ones (**Table 3**). Adverse effects of salt stress on plant growth are mainly attributed to limited water uptake as a result of ion osmotic effect, which in turn affect the photosynthetic rate, cell function, nutrient balance and several other metabolic functions (Kumar et al., 2005). In the present study, it seems likely that SAT-17_rif_ modulated the plants’ ability to uptake water more efficiently, probably by modulating the root system architecture as the root hairs and lateral roots are the main sites for PGPR colonization in members of the family Poaceae (Combes-Meynet et al., 2011; Couillerot et al., 2011). Moreover, PGPR supply may enhance nutrient uptake by stimulating root formation (Yildirim et al., 2011) and inhibiting the salt-ion accumulation (Mayak et al., 2004), which in turn promotes plant growth (Supplementary Figure S3). Furthermore, PGPR inoculation might altered the source-sink relations as *Capsicum annuum* plants, co-inoculated with *Azospirillum brasilense* and *Pantoea dispersa*, exhibited the higher plant dry matter accumulation associated with a higher source activity, stomatal conductance and photosynthesis (del Amor and Cuadra-Crespo, 2012). As the SAT-17 produced optimum IAA *in vitro*, it was suggested that IAA acted as a negative feedback signal to temporarily repress cytokinin synthesis in the roots and their transport to the shoot (Rahayu et al., 2005) leading to increased root elongation. The observed effects on plant biomass may also be attributed to modulated regulation of ethylene, as SAT-17 exhibited significant ACC deaminase activity (Dodd and Pérez-Alfocea, 2012; Kim et al., 2014). Our results coincide well with the findings of Rajput et al. (2013) who reported an increase in the growth and yield of wheat under salt stress conditions after treating the wheat plants with *Planococcus rifietoensis* strain SAL-15. In another study, Ahmad et al. (2014) reported that the integrated use of PGPR, biogas slurry and chemical nitrogen enhanced maize growth and productivity. The PGPR strains *Erwinia persicinus* RA2 (with ACC deaminase activity) and *Bacillus pumilus* WP8 (without ACC deaminase activity) significantly enhanced tomato growth and quality under seawater irrigation (Cha-Um and Kirdmanee, 2009).

In non-inoculated plants, salt stress increased the MDA content and triggered the production of H_2_O_2_; this is an indication of oxidative damage to membranes, as reported for various crops (Cui and Wang, 2006; Nasraoui-Hajaji et al., 2012). Increased MDA contents at the cellular level resulted in oxidative injury as well as a disruption of nutrient ion balance (Azooz et al., 2009). Ashraf (2010) reported that reduced leaf water potential was observed due to lipid peroxidation and ionic leakage induced by salt stress. Inoculation of maize seedlings with *S. sciuri* SAT-17_rif_ significantly prevented salt-induced lipid peroxidation of membranes, as evidenced by the decreased MDA contents in the present study.

Proline may act as an enzyme protectant and stabilize the structure of other macromolecules (Mahajan and Tuteja, 2005).

In the present study, increased proline accumulation in the inoculated plants alleviated the adverse effects of salt stress. It is well known that proline accumulation and other compatible solutes under stress conditions might help with the osmotic adjustment at a cellular level. PGPR are well known for producing resistance in plants against various stresses like salinity through the enhanced production of antioxidants (Younesi and Moradi, 2014).

The adverse environmental conditions triggered the synthesis of hydrogen peroxide (H_2_O_2_), superoxide (O_2_^-^), and/or hydroxyl (OH^-^) radicals (Shigeoka et al., 2002); all of these ROS pose potential hazards if they are not detoxified by the plants. The anti-oxidative defense system, comprised of enzymatic and non-enzymatic components, provides a mechanism against the deleterious actions of ROS in plant cells (Ali and Ashraf, 2011). Inoculated maize plants under salinity treatments (75 and 150 mM) showed significantly reduced ROS levels and elevated levels of antioxidant enzymes (CAT, POD), suggesting the induction of oxidative damage repair mechanisms (**Table 4**). Our results were in agreement with earlier findings that PGPR strains under salinity stress environments triggered the stress responsive non-enzymatic (Kim et al., 2014; Islam et al., 2015) and enzymatic anti-oxidative defense systems (comprised of CAT and POD) (Ali and Ashraf, 2011; Masood et al., 2012). The PGPR-induced salt alleviation may be attributed to bacterial exopolysaccharides which adhere to soil Na^+^ and prevent its transfer to shoots/leaves (Ashraf et al., 2004). In addition, PGPR also confer salt-tolerance in plants by the tissue-specific regulation of the Na transporter *HKT1* (Zhang et al., 2008). Under saline conditions, PGPR regulate the enzymes of important metabolic pathways (tricarboxylic acid cycle, glyoxylate cycle, glycolysis) and improved the energetic status of the plant (Sheng et al., 2011), which in turn help to sustain ionic homeostasis by maintaining the Na^+^ exclusion capacity in the roots and delay the incidence of toxic ionic effects (Pérez-Alfocea et al., 2010). Sahoo et al. (2014) reported that *Azotobacter vinellandii* (SRI*Az*3), isolated from rice rhizosphere, improved rice productivity by inducing stress tolerance and altering plant endogenous hormonal levels.

Salt stress negatively affected the uptake of N, P and K in the roots and shoots of maize, probably due to the increased uptake of Na and Cl ions (**Table 5**). Earlier works reported that K^+^ uptake in tomato plants decreased due to the antagonistic relationship of Na^+^ and K^+^ at the root surface under NaCl stress (Bastias et al., 2010). Furthermore, the root membrane structure and selectivity for ions has also been reported to be negatively affected under salt stress due to the interference of Na^+^ with K^+^ (Grattan and Grieve, 1999). Inoculation of maize seedlings with *S. sciuri* SAT-17_rif_ significantly increased the uptake of N, P, and K^+^ in roots (and translocation to the shoots) compared to the non-inoculated control. *S. sciuri* SAT-17 was a potential phosphate-solubilizing strain; hence it enhanced the mobilization of P from the soil to the roots/shoots. Similarly, the plant growth-promoting effect of the strain favored the maize plants accumulating more macronutrients in the biomass. Our results are in accordance with the findings of Mohamed and Gomaa (2012), who reported a significant increase in macronutrient (N, P, K^+^, Ca^2+^ and Mg2^+^) concentrations in radish when the seeds were inoculated with PGPR (*Bacillus subtilis* and *Pseudomonas fluorescens*). Yildirim et al. (2011) reported that the increased nutrient concentrations in broccoli plants were due to the increased root surface area as well as root exudation. The enhanced root exudation up-regulates microbial activity, leading to increased soil nutrient solubility and, later, higher influx into the plant roots (Adesemoye et al., 2008). The enhanced nutrient uptake by maize roots, after SAT-17 inoculation, may be attributed to a low rhizospheric pH which increased the bioavailable fraction of cationic nutrients as reported earlier (Abou-Shanab et al., 2006). However, the underlying mechanisms are not yet completely elucidated. A similar trend was also determined for total chlorophyll and protein contents of the plants. Earlier works with PGPR have also been shown to alleviate NaCl stress by increasing the leaf chlorophyll contents. The increase in leaf chlorophyll contents under PGPR inoculation was attributed to the increased nutrient availability (Karlidag et al., 2013). The observed effect is also likely due to root proliferation and enhanced water absorption, which in turn increases the leaf numbers and leaf surface area for photosynthesis. Hence, increased chlorophyll contents, after *S. sciuri* SAT-17_rif_ inoculation, were responsible for the shoot growth enhancement under salt stress which offered new binding sites for nutrient ions. Under salt stress, the protein contents were significantly reduced, probably due to biological degradation, as the toxic ions can activate the mechanism of protein denaturation in maize plants (Gadd and Griffith, 1978). Alterations in protein contents under saline conditions may modulate the enzyme activities responsible for the anti-oxidative defense to cope with salt-mediated production of ROS (Hossain et al., 2012). Inoculation of maize seedlings with *S. sciuri* SAT-17_rif_ significantly increased the protein contents of maize, enabling the plants to synthesize anti-oxidative enzymes and better withstand the salt stress.

## Conclusion

The salt-tolerant rhizobacterium *S. sciuri* strain SAT-17, characterized in the current study, exhibited substantial phosphate solubilization as well as indole-3-acetic acid production and 1-aminocyclopropane-1-carboxylic acid deaminase activity. The inoculation of maize with SAT-17 improved plant growth alongside decreasing the reactive oxygen species levels and increasing the cellular antioxidant enzyme activities (CAT, POD and proline). Moreover, the inoculation increased the uptake of N, P and K to maintain optimum nutrient, chlorophyll and protein levels in maize plants, thereby preventing drought-induced lipid peroxidation of membranes under salt stress. It has been concluded that the use of *S. sciuri* SAT-17 could serve as an efficient approach for enhancing crop tolerance to salinity in arid and semi-arid regions. This study opens the future directions for researchers to investigate about the genetic mechanism involved in salinity tolerance induction by *S. sciuri* SAT-17 in maize. The study on expression profiling of some stress-responsive genes of maize in response to *S. sciuri* SAT-17 inoculation might be helpful to understand about molecular cross-talk between plant and bacterial strain.

## Author Contributions

MSA: designed experiments, data analysis, manuscript preparation. MT: designed experiments, manuscript preparation. MS: designed experiments, manuscript preparation, data analysis. MA: manuscript preparation, data analysis. MTJ: critical revision of manuscript, data analysis. SS: student who practically performed experiments, manuscript preparation. SR: student who practically performed experiments, manuscript preparation.

## Conflict of Interest Statement

The authors declare that the research was conducted in the absence of any commercial or financial relationships that could be construed as a potential conflict of interest.
