# Supplementary material for: Deciphering Staphylococcus sciuri SAT-17 Mediated Anti-oxidative Defense Mechanisms and Growth Modulations in Salt Stressed Maize (Zea mays L.)
Source: Front Microbiol. 2016 Jun 9;7:867. doi: 10.3389/fmicb.2016.00867 (PMC4899454; doi:10.3389/fmicb.2016.00867)
Supplement: Supplementary file 1 [file Data_Sheet_1.DOCX]

**Supplementary figure** 1. Multiple sequence alignment of 16S rRNA gene of *S. sciuri* SAT-17 and closest GenBank matches

**Supplementary figure 2.** Pure culture of *S. sciuri* SAT-17 on agar plate

**Supplementary figure 3.** Effect of *S. sciuri* SAT-17 inoculation on maize growth at different salt levels
