# Supplementary figures and images for: Deciphering Staphylococcus sciuri SAT-17 Mediated Anti-oxidative Defense Mechanisms and Growth Modulations in Salt Stressed Maize (Zea mays L.)
Source: Front Microbiol. 2016 Jun 9;7:867. doi: 10.3389/fmicb.2016.00867 (PMC4899454; doi:10.3389/fmicb.2016.00867)

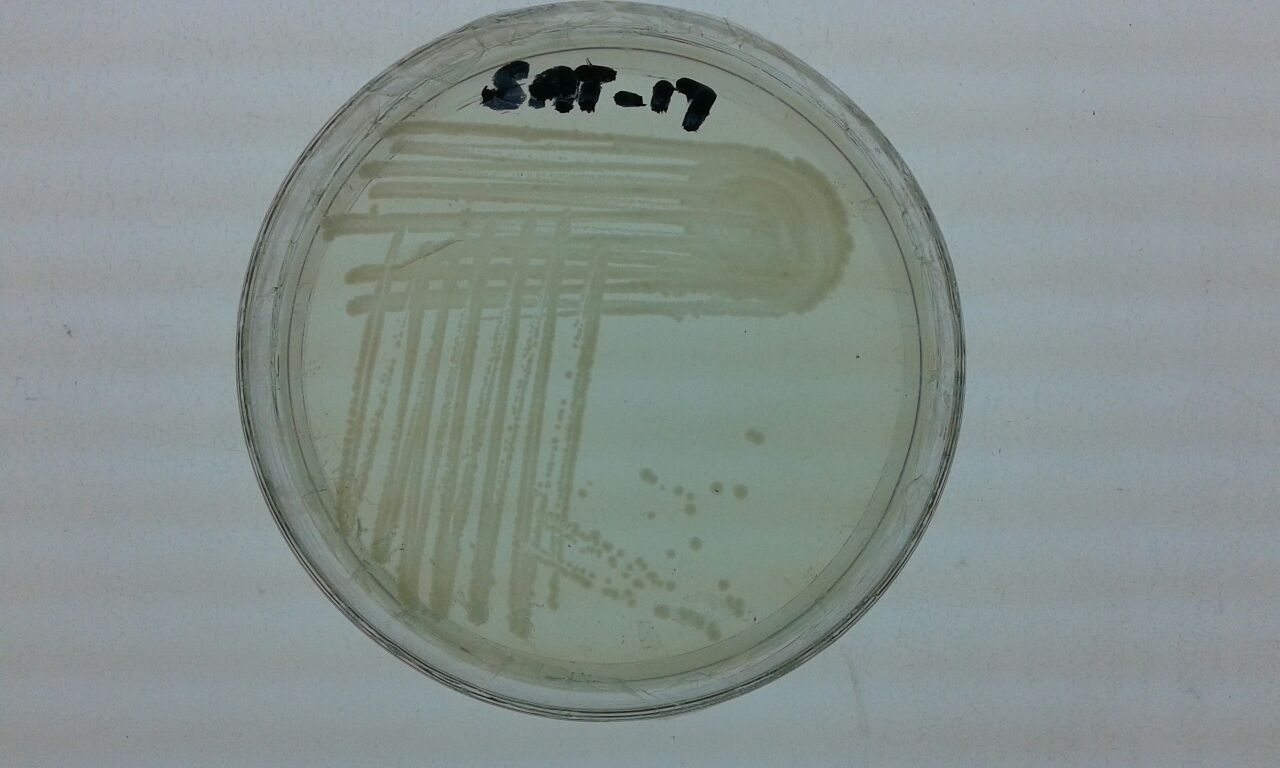

Supplement: Supplementary file 3 [file Image_2.JPEG]

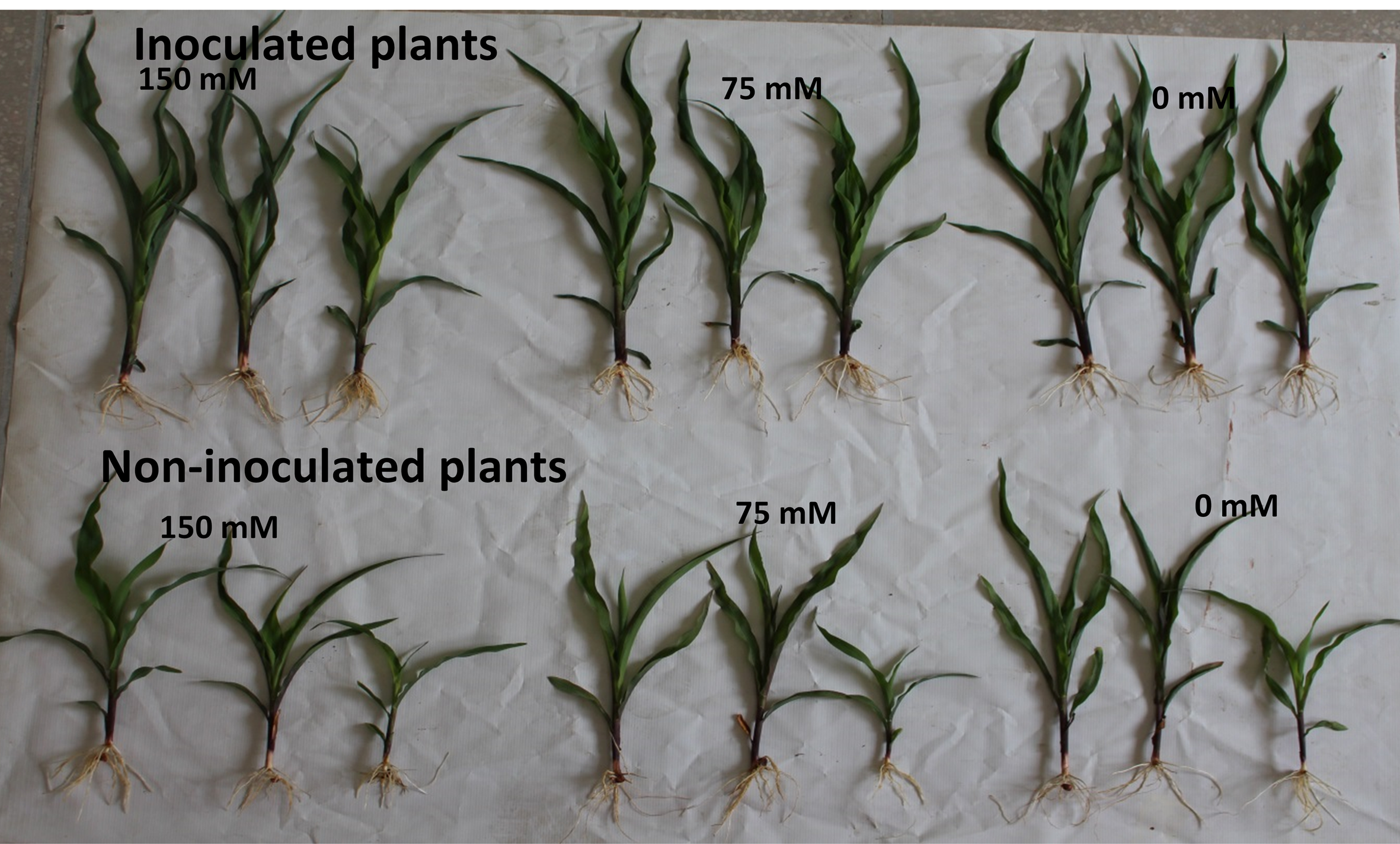

Supplement: Supplementary file 4 [file Image_3.TIF]
